# MYCN-mediated transcriptional repression in neuroblastoma: the other side of the coin

**DOI:** 10.3389/fonc.2013.00042

**Published:** 2013-03-11

**Authors:** Samuele Gherardi, Emanuele Valli, Daniela Erriquez, Giovanni Perini

**Affiliations:** ^1^Department of Pharmacy and Biotechnology, University of BolognaBologna, Italy; ^2^Health Sciences and Technologies – Interdepartmental Center for Industrial Research University of BolognaBologna, Italy

**Keywords:** MYCN, neuroblastoma, transcriptional repression, cell differentiation, apoptosis, cell cycle

## Abstract

Neuroblastoma is the most common extra cranial solid tumor in childhood and the most frequently diagnosed neoplasm during the infancy. MYCN amplification and overexpression occur in about 25% of total neuroblastoma cases and this percentage increases at 30% in advanced stage neuroblastoma. So far, MYCN expression profile is still one of the most robust and significant prognostic markers for neuroblastoma outcome. MYCN is a transcription factor that belongs to the family of MYC oncoproteins, comprising c-MYC and MYCL genes. Deregulation of MYC oncoprotein expression is a crucial event involved in the occurrence of different types of malignant tumors. MYCN, as well as c-MYC, can heterodimerize with its partner MAX and activate the transcription of several target genes containing E-Box sites in their promoter regions. However, recent several lines of evidence have revealed that MYCN can repress at least as many genes as it activates, thus proposing a novel function of this protein in neuroblastoma biology. Whereas the mechanism by which MYCN can act as a transcriptional activator is relatively well known, very few studies has been done in the attempt to explain how MYCN can exert its transcription repression function. Here, we will review current knowledge about the mechanism of MYCN-mediated transcriptional repression and will emphasize its role as a repressor in the recruitment of a precise set of proteins to form complexes capable of down-regulating specific subsets of genes whose function is actively involved in apoptosis, cell differentiation, chemosensitivity, and cell motility. The finding that MYCN can also act as a repressor has widen our view on its role in oncogenesis and has posed the bases to search for novel therapeutic drugs that can specifically target its transcriptional repression function.

## INTRODUCTION

Neuroblastoma is one of the most frequent extracranial solid tumor in childhood. It arises from the neural crest cells during development of the sympathetic nervous tissue. The overall incidence is approximately one case in 7,000 live births and the median age at diagnosis is about 18 months. Neuroblastoma is responsible for around 15% of all pediatric oncology deaths. Roughly 25% of most aggressive neuroblastomas are characterized by the amplification/overexpression of the MYCN transcription factor that nowadays is considered one of the most robust prognostic factors for the neuroblastoma unfavorable outcome (Bordow et al., 1998; Cohn et al., 2009). Recently it has been demonstrated that MYCN protein level is predictive for neuroblastoma outcome independently from its genomic amplification and up-regulation (Chan et al., 1997). In fact, it is well known that MYCN protein level is not always correlated with its mRNA level thus suggesting possible different ways to increase MYCN protein stabilization. Indeed, activated PI3K/AKT pathway and Aurora kinase A activity (AURKA) has been demonstrated to be involved in MYCN protein stabilization (Kenney et al., 2004; Chesler et al., 2006; Otto et al., 2009; Segerstrom et al., 2011).

Another formal demonstration that gene and protein expression are not always equivalent came from Molenaar et al. (2012). They found out that MYCN protein level can be enhanced by LIN28B overexpression through its repression activity on Let-7 microRNA (miRNA) which regulates MYCN protein amount (Molenaar et al., 2012).

Furthermore, Valentijn et al. (2012) have identified a novel MYCN-dependent signature consisting of 157 genes that directly correlate with MYCN protein level but not with MYCN amplification thus increasing the MYCN protein level significance in neuroblastoma development and outcome. Notably, among these genes there are 21 down-regulated genes involved in neuronal differentiation (Valentijn et al., 2012).

MYCN, as a member of Myc family, is a transcription factor primarily known for its transactivating function, but during the last 10 years it has been also shown that it has the ability to repress transcription of target genes. The aims of this review are to discuss the role of MYCN-mediated repression in neuroblastoma onset and summarize the knowledge so far accrued on the molecular mechanism(s) by which MYCN can exert transcriptional repression on a specific subset of genes, the majority of which involved in apoptosis, cell differentiation, and cell cycle regulation.

## MYCN

MYCN was first discovered in 1983 by Schwab et al. (1983) as a paralog of the most popular c-Myc (Vennstrom and Bishop, 1982; Vennstrom et al., 1982). The Myc family of transcription factors is composed by three elements: c-MYC, L-MYC, and MYCN. The Myc oncoproteins are transcription factors belonging to a subset of the larger class of proteins containing basic-region/helix-loop-helix/leucine-zipper (BR/HLH/LZ) motifs. They are structurally similar: the N-Term region can interact with co-activators or co-repressors and contains several domains conserved among the Myc family members, whereas the C-Term carries a BR/HLH/LZ domain required for dimerization with the partner MAX and for interaction with DNA.

MYCN is predominantly expressed in the peripheral and central nervous systems, lung, kidney, and spleen during embryonic development, and it is subjected to a strict temporal and spatial expression pattern as shown by comparison of fetal and adult brain cells (Grady et al., 1987) and by analyses of fetal mouse tissues during the development (Jakobovits et al., 1985; Zimmerman et al., 1986). MYCN heterodimerizes with MAX forming a functional transcriptional activator that can bind DNA upon a specific consensus sequence CACGTG called E-Box. Like c-MYC, MYCN recruits histone acetyltransferase complexes [i.e., transformation/transcription domain-associated protein (TRRAP) and Tat-interactive protein 60 kDa (TIP60)] that keep chromatin in an active state (Frank et al., 2003). Moreover, it was demonstrated that MYC can also promote transcript elongation by recruiting positive transcription elongation factor (pTEF-β) that induces phosphorylation of Ser2 of RNA polymerase C-terminal domain (CTD; Majello et al., 1999).

First indication that MYC can act as a transcriptional repressor came from Cleveland et al. (1988). Nonetheless it was not possible to identify specific DNA sequences that were bound by MYC in order to enact transcription repression. Later on, these issues were elucidated demonstrating that MYC induces transcriptional repression by an indirect binding to DNA trough interaction with basal transcription factors as specific protein 1 (SP1; Gartel et al., 2001) or Myc-interacting zinc-finger protein-1 (MIZ-1; Peukert et al., 1997). The importance of MYC induced repression were then emphasized by genome-wide chromatin immunoprecipitation analyses which demonstrated that more than 40% of MYC binding sites lack recognizable E-Box sequences (Zeller et al., 2006) suggesting that MYC can repress many target genes, most likely through mechanisms that are distinct from those used to activate transcription.

We already mentioned in this review that MYCN and c-MYC are highly homologous and share several domains including the transactivating and DNA binding domains. This has led to think that c-Myc and MYCN can function in similar fashion although expressed in different cellular backgrounds. Thus, several investigations pertaining to MYCN activity have been, somehow, suggested by previous studies on c-Myc. More specifically, these studies revealed that not all genes are repressed by Myc through the same mechanism.

Some Myc repressed target genes contain a subclass of initiator elements (INRs consensus, YYCAYYYYY, where Y represents a pyrimidine base T/C), which are usually, but not exclusively, present in TATA-less promoter types. INRs elements are recognized by transcription factor II D (TFII-D) as well as a number of regulatory proteins like TFII-I, YY1, and the MIZ-1.

It has been demonstrated that c-Myc can interact with MIZ-1 and that the MIZ-1/Myc complex promotes stabilization of Myc by inhibiting its ubiquitination and degradation (Park et al., 2001; Eilers and Eisenman, 2008; Akter et al., 2011). MIZ-1 (also known as ZBTB17) gene encodes for a protein of 721 aa characterized by a series of consecutive 13 zinc-finger domains (N-terminus) and a BTB (BR-C, ttk, and bab)/POZ (pox virus and zinc-finger) domain which is a protein/protein interaction domain found in a multiple zinc-finger proteins. MIZ-1 interacts with Myc “outside” the helix-loop-helix (HLH) domain, but does not interact with Mad, Max, and Mnt (Peukert et al., 1997; Eilers and Eisenman, 2008). Nowadays the putative mechanism of MYCN-mediated repression through interaction with MIZ-1 is still unclear.

Other sets of genes repressed by Myc do not contain INR sequences and the repression appears to be mediated by group-specific component (GC)-rich regions that are recognized by other factors. An important GC binding protein that is involved in the repression mechanism is the basal transcription factor 1 (SP1; Liu et al., 2007; Marshall et al., 2010, 2011; Valli et al., 2012). SP1 is a zinc-finger protein of 785 aa, involved in many cellular processes including: differentiation, growth, apoptosis, responses to DNA damage, and chromatin remodeling. It possesses two transcriptional activation domains (TADs) and normally recruits TATA-binding protein (TBP). The interaction between MYCN and SP1 was deeply investigated by Iraci et al. (2011) that identified, through GST pull down assay, the MB2 (MYC box 2) as the MYCN domain responsible of its interaction with SP1. Once MYCN is bound to SP1, it exerts its repressive function via recruitments of chromatin modifiers such as histone deacetylases. In 2007, Marshall and colleagues have also shown that MYCN can repress transcription of the transglutaminase 2 (TG2) gene through interaction with SP1 and subsequent recruitment of histone deacetylase 1 (HDAC1) that removes the acetyl group of histone tail inducing a greatercompacting of chromatin and consequent transcriptional repression (Liu et al., 2007). Importantly, chromatin immunoprecipitation studies have shown that HDAC1 recruitment by MYCN occurs in absence of the partner MAX and can be disrupted using trichostatin A (TSA), an HDAC1 inhibitor. However, while HDAC1 is released by the complex in the presence of TSA, MYCN remains associated with the TG2 promoter suggesting that the formation of a SP1/MYCN/HDAC1 complex is quite dynamic. MYCN as well as c-MYC seems to interact with several paralogs of HDAC1 such as HDAC2 or HDAC3 (Zhang et al., 2012a, b). Marshall et al. (2010) demonstrated that MYCN can inhibit the transcription of CyclinG2 gene through the interaction with SP1 and HDAC2 and consequent transcriptional repression of the gene. *In vitro* analyses of the MYCN regions required for its interaction with both SP1 and MIZ-1 show that MYCN MB2 domain can directly interact with SP1, while the basic helix-loop-helix leucine-zipper (bHLHZip) domain is required for interaction with MIZ-1. The “ternary complex” can also drive the transcriptional repression of genes such as TRKA (tyrosine kinase receptor A), P75NTR (p75 neurotrophin receptor), and p21 in neuroblastoma by recruitment of HDAC1 on the respective promoters (Iraci et al., 2011).

Finally, a MYCN/SP1 complex also appears to be critical for the recruitment of SIRT1 (a NAD-dependent histone deacetylase; Marshall et al., 2011) to repress the transcription of MPK3 gene. In this case the MB1 domain of MYCN encompassing amino acids 1–88 is required for its physical association with SIRT1 (Liu et al., 2007).

## CELL CYCLE AND PROLIFERATION

Although MYCN and c-MYC are generally defined as oncogenes which act as positive transcriptional regulators of pro-proliferative gene networks, it has been also proposed that they may also promote the oncogenic process through repression of target genes including both protein-encoding genes and miRNAs (Shohet et al., 2011). Like c-MYC, MYCN, when induced ectopically, stimulates the re-entry of quiescent cells into the cell cycle and shortens the time taken to progress through the cell cycle, specifically reducing the G1 phase and decreasing cell attachment to the extracellular matrix. Nevertheless, reduction of MYCN expression level promotes cell cycle arrest, differentiation, and apoptosis (Bell et al., 2010). Cyclin-dependent kinase inhibitors (CKIs) are a fundamental class of proteins that negatively regulate progression through the cell cycle and so prevent uncontrolled cell growth and cancer. There are two distinct families of CDK inhibitors: INK4 and Cip/Kip (Sherr and Roberts, 1999). The members of the INK4 family (p16^INK4a^, p15^INK4b^, p18^INK4c^, p19^INK4d^) specifically inhibit the activity of CDK4 and CDK6, whereas Cip/Kip members (p21^CIP1/WAF1^, p27^KIP1^, p57^KIP2^) inhibit all the other cyclin–CDK complexes. MYCN represses the expression of p21^CIP1^, by forming a complex with transcriptional regulators, such as the MIZ-1 and SP1 (Iraci et al., 2011) thereby promoting cell growth and cancer onset. Because of its high homology with c-MYC, it is plausible to think that MYCN may repress p15^INK4b^ as well, through the same mechanism enacted by c-MYC, although this has not been formally demonstrated (Staller et al., 2001; Wu et al., 2003).

A more complex scenario of the role of MYCN as a pivotal regulator of the cell cycle is provided by studies employing small interfering RNAs (siRNAs). MYCN knock-down in neuroblastoma MYCN-amplified (MNA) cell lines such as IMR32 and SKNBE(2c) determines an up- or down-regulation of several cell cycle related genes involved in different important signaling pathways. For example, Bell et al. (2007) have identified a number of genes involved in regulation of the G1 checkpoint that are differentially expressed after MYCN knock-down. Tumor protein 53-induced protein 1 (TP53INP1) has been reported to cause a G1 arrest and apoptosis and its expression level increases after MYCN knock-down. This is a MYCN-dependent effect as confirmed by experiment in TET21N neuroblastoma cells that carry a MYCN conditional minigene. TP53INP1 regulates p53 and p73 transcriptional activity, and in particular has been found to increase p53-dependent p21^WAF1^ transcription (Bell et al., 2007). A member of Dickkopf (DKK) family, DKK1, is down-regulated by MYCN in neuroblastoma and this might contribute to the well-documented stimulation of cell proliferation by MYCN (Lutz et al., 1996). Surprisingly, DKK1 inducible expression did not inhibit the canonical Wnt/β-catenin signaling, suggesting a role of DKK1 in an alternative route of the Wnt pathway (Koppen et al., 2007).

Transforming growth factor β (TGF-β) is a potent inhibitor of cell proliferation and induces differentiation and growth arrest in certain cell types. TGF-β signaling pathway targets include bone morphogenetic proteins, Smad transcription factors and activins. p57^cip2^, a CDK inhibitor that targets cyclin D–CDK4/6 complexes, is the downstream transcriptional target of TGF-β signaling which causes a G1 arrest. Levels of TGF-β2 and p57^cip2^ expression increased 48 h after MYCN knock-down in p53wt IMR32 cells. Therefore, amplification of MYCN expression in this neuroblastoma genetic background may repress TGF-β signaling in order to prevent cyclin D inhibition by p57^cip2^ (Bell et al., 2007).

Valentijn et al. (2005) suggest that MYCN expression represses cell division cycle 42 gene (CDC42) a G-protein involved in a cytoskeletal remodeling pathway. Ectopic MYCN expression decreased CDC42 expression in the Tet21N system and conversely MYCN siRNA increased CDC42 expression (Valentijn et al., 2005).

All MYCN downstream regulated gene (NDRG) family members seem to be required in many biological responses even if their exact role is not yet clear. NDGR proteins contribute to cell proliferation, differentiation, development, and stress responses. Emerging evidence suggests that mutations in these genes are associated with diverse neurological and electrophysiological syndromes. Shimono et al. (1999) reported NDRG1 to be down-regulated by Mycn in mice. Murine NDRG1 promoter activity is repressed by Mycn and Myc (Shimono et al., 1999). Human NDRG1 was also found to be down-regulated in MYCN-amplified neuroblastoma cell lines by interaction of the MYCN protein with the NDRG1 core promoter (Li and Kretzner, 2003). Expression of human NDRG2 is down-regulated by MYCN via transcriptional repression via binding of the MIZ-1 at the core promoter (Zhang et al., 2006). Furthermore, the repression of the NDRG1 and NDRG2 promoter activity by MYC is sensitive to TSA, indicating involvement of histone deacetylase activity in the mechanism of transcriptional repression of these promoters (Shimono et al., 1999; Zhang et al., 2006).

## CELL INVASION

As the majority of aggressive solid tumors, neuroblastoma cells develop the ability to invade the surrounding tissues from the primary localization. Once cancer cells reached blood vessels or the lymphatic system, they metastasize throughout all the body. MYCN plays a central role in neuroblastoma invasiveness primarily by direct or indirect repression of specific target genes. Judware and Culp (1997) demonstrated that MYCN overexpression could alter the cell–matrix and cell–cell interactions by reducing expression of α2, α3, β1 integrin subunits. Caveolin-1 is also directly repressed by MYCN (Park et al., 2001) and its down-regulation elicits anchorage-independent growth and tumor formation (Galbiati et al., 1998).

Intriguingly, MYCN directly regulates transcription of a specific subset of the ATP-binding cassette (ABC) transporters genes that in addition to their typical drug efflux function appear to control cell motility and invasion (Porro et al., 2010) through mechanism(s) that are not yet known (Henderson et al., 2011).

Over the last 10 years, a lot of effort has been made on the biology of miRNAs. MYCN may also activate the transcription of many miRNAs thus causing the indirect repression of genes regulated by them, and interfering, among other things, with the activity of the cell adhesion pathway (Ma et al., 2010; Mestdagh et al., 2010).

## ANGIOGENESIS

Angiogenesis is a physiological phenomena consisting in the generation of new capillaries from preexisting vessels. It occurs mainly during embryonic development but can also occur in the adult, for example, during the wound healing and in granulation tissue. Furthermore, angiogenesis is a key feature of more aggressive solid tumors, indeed through the formation of new capillaries to the tumor mass ensures the continuous flow of nutrients and the ability to metastasize. Molecularly, angiogenesis is the result of a complex and strictly regulated interplay between humoral stimulators and inhibitors (Carmeliet and Jain, 2011). Among activators, the vascular endothelial growth factor (VEGF) family holds the most important role (Nagy et al., 2007; Ferrara, 2009), but angiogenesis is also induced by other active molecules such as those encoded by the fibroblast growth factor (FGF) family (Beenken and Mohammadi, 2009). Substantially, these two families of activators can induce all the necessary step for a complete angiogenesis.

Angiogenesis is also a key pathological marker in neuroblastoma (Katzenstein et al., 2000; Ribatti et al., 2004) and many works correlate its induction to the amplification and/or overexpression of MYCN transcription factor (Ribatti et al., 2002; Kang et al., 2008). Intriguingly, MYCN has both the ability to transcriptionally activate angiogenic factors and to represses directly the transcription of angiogenic inhibitors. One of the first evidences of MYCN-repressed inhibitors was shown by Fotsis et al. (1999). They purified a protein factor from non-MYCN-amplified neuroblastoma cells culture medium; such a peptide was absent in MYCN-amplified culture medium and exhibited anti-angiogenic properties (Fotsis et al., 1999). A couple of years later, the same authors identified this peptidic factor as Activin-A (Breit et al., 2000). By the same strategy, Hatzi et al. (2002) identified IL-6 as another important anti-angiogenic factor repressed by MYCN.

## REPRESSION OF PRO-APOPTOTIC GENES

In addition to that previously described another mechanism by which MYCN can contribute to neuroblastoma onset is through the repression of nerve growth factor receptor (NGFR) gene. NGFR, also known as P75NTR, encodes a membrane receptor that binds neurotrophins with low affinity. The role of NGFR in neuroblastoma is still unclear. However, some lines of evidence suggest that the intracellular regions of NGFR containing death domains might send signals to induce neuronal cell death and NGFR expression level are prognostic in neuroblastoma correlating with undifferentiated tumors (Casaccia-Bonnefil et al., 1998; Schulte et al., 2009). As for TRKA, the expression of NGFR is strongly down-regulated in aggressive neuroblastoma having MYCN overexpression. Recently, Iraci et al. (2011) demonstrated that MYCN can bind NGFR promoter thus repressing its expression. They also proved that MYCN silencing using a siRNA technology induces a NGFR re-expression and sensitize neuroblastoma cells to NGF-mediated apoptosis (Iraci et al., 2011).

Intriguingly, MYCN also represses genes characterized by an anti-apoptotic function such as Galectin-3 suggesting a very complex balance on apoptosis regulation (Veschi et al., 2012).

## DIFFERENTIATION

Differentiation is a cellular process by which a less specialized cell becomes more specialized during development. Up to date, cellular differentiation is very important in cytopathology where the level of differentiation is used as a measure of cancer progression and aggressiveness. In fact, more aggressive neuroblastoma stages (III and IV) are usually characterized by a low grade of differentiated cells. Is well known that MYCN can form several complexes capable of regulating both directly and indirectly a set of genes involved in neuronal differentiation processes. High level of MYCN drives down-regulation of NLRR3, a gene with effects on cell differentiation (Koppen et al., 2007; Akter et al., 2011). Jiang et al. (2011) suggest that a stable MYCN knock-down using lentiviral short hairpin RNAs (shRNAs) can induce p27 and nuclear export sequence (NES) increase with subsequent stimulation of the neuronal differentiation pathways. In addition, neuroblastomas without MYCN amplification are characterized by good expression levels of Shh–GLI1–Ptch1 and good prognosis though the molecular link between these two aspects is unknown (Souzaki et al., 2010). In contrast to other cancer types, the Hh pathways may be associated with commitment and differentiation stimuli in neuroblastoma (Ahlgren and Bronner-Fraser, 1999; Williams et al., 2000). Moreover MYCN has been found to play a pivotal role in neurotrophic tyrosine receptor kinase (NTRK) gene family regulation especially on TRKA receptor. Is well known that MYCN expression counter-correlates with TRKA expression and that more aggressive neuroblastomas are characterized by low levels of TRKA (Nakagawara et al., 1992, 1993; Suzuki et al., 1993; Brodeur, 2003). Nara et al. (2007) found out that after silencing MYCN using RNA interference (RNAi) technology the relative expression of TRKA and TRKC were significantly up-regulated; in addition, Iraci et al. (2011) described the molecular mechanism by which MYCN can directly repress TRKA expression. Furthermore, MYCN can up-regulate Bmi1t protein thus leading to repression of KIF1Bb and tumor suppressor in lung cancer 1 (TSLC1) transcription and maintaining an undifferentiated cell status (Ochiai et al., 2010). Another important contributor to cell differentiation is TG2. TG2 is a multifunctional enzyme that catalyses transamidation and multimerization of proteins. It is involved in both intra- and extracellular processes and its deregulation determine various downstream effects in several types of cancer. It has been demonstrated that the MYCN-mediated repression of TG2 is essential to inhibit neuronal differentiation in MYCN overexpressing neuroblastoma cells. MYCN recruits HDAC1 protein to a core promoter of TG2 gene containing SP1 binding sites where SP1 transcription factor is bound (Fesus and Piacentini, 2002; Lorand and Graham, 2003; Liu et al., 2007).

Finally, a recent study by Valli et al. (2012) has provided support that MYCN can prevent neuronal differentiation by repressing transcription of the CDKL5 gene through interaction with SP1.

## miRNA AND MYCN: AN INDIRECT MECHANISM OF GENE EXPRESSION CONTROL

Almost 20 years ago, Victor Ambros and colleagues found out that LIN-14 protein abundance, in *C. elegans*, was directly regulated by a short RNA product encoded by the lin4 gene (Lee et al., 1993). In the last 20 years, hundreds of studies have addressed short RNA function and regulation. miRNA have been found to be misregulated in a variety of tumors and some of them have a tumor suppressor function while others have a oncogenic function (oncomiR). Numerous studies in neuroblastoma have shown that MYCN can act as a transcriptional regulator factor even on miRNA expression. MYCN could suppress miR-152 expression thus playing an important role in the control of the genome methylation status, DNA methyltransferase 1 (DNMT1) being a direct target of miR-152 (Das et al., 2010). A well-known target of MYCN is the miR-17-92 cluster. Indeed, MYCN up-regulates miR-17-92 cluster and one of the effects is a down-regulation of DKK3 a gene with tumor suppressor function involved in Wnt pathway and having a significance value in neuroblastoma prognosis (De Brouwer et al., 2012). Low levels of DKK3 are usually associated with MYCN-amplified tumors and its down-regulation promotes G1 arrest checkpoint skipping through its negatively regulation of β-catenin and cyclin D. The miR-17-92 cluster has been found to be involved even in the negative regulation of cluster in a gene involved in metastasization process (Chayka et al., 2009). Besides miR-591, a short tumor suppressor RNA, is down-regulated in neuroblastomas with MYCN amplification (Shohet et al., 2011). Interestingly, miR-542-5p is another miRNA found expressed at low level in patients with highly aggressive neuroblastoma. It is still unknown if MYCN directly or indirectly regulates this miRNA but it is clear that miR-542-5p expression correlates with TRKA expression both *in vivo* and *in vitro* thus playing a role in neuroblastoma outcome (Schulte et al., 2010). Recently, Lynch et al. (2012) have found that MYCN can bind at promoter level of miR-335 promoting its repression, resulting in up-regulation of TGF-β with consequent enhancement of cell migration and invasiveness.

Finally MYCN up-regulates a set of miRNA with oncogenic function (oncomiR) as miR380-5p, miR-9, and miR-221 but the molecular mechanism is still unknown (Schulte et al., 2008; Ma et al., 2010; Swarbrick et al., 2010). On top of this it is well taken that MYCN overexpression could be associated with genomic instability and that such an instability affect miRNA expression profiles (Shohet et al., 2011).

## DISCUSSION AND PERSPECTIVE

Nowadays it is well established that MYCN plays a pivotal role in neuroblastoma tumorigenesis. MYCN, as well as c-MYC, was widely studied as a transcriptional activator leading to the conclusion that up-regulated target genes are responsible for many aspects of the tumor malignancy. On the other hand, a significant amount of work has been done to better understand the importance of MYCN-mediated transcriptional repression in neuroblastoma. From the data available so far in the scientific literature we could conclude that MYCN-mediated transcription repression is at least important as transcription activation. MYCN has the ability to activate genes that increase the malignancy of the tumor and at the same time has the ability to repress genes that can prevent tumor formation or at least that can keep the tumor restricted to a more benign form. One interesting aspect that emerges from these studies is that MYCN can directly interact with many chromatin components, particularly transcription factors and histone modifiers. These findings reveal a complex, still incomplete scenario suggesting that Myc-mediated transcriptional repression needs further investigation and deserves to be considered as a genuine and critical function exerted by this transcription factor during oncogenesis. When we think of Myc as a transcription activator we always see it as part of a long lasting partnership with Max. It is this specific heterodimer that does the job for activated transcription. Very recent studies have shown that this complex is simply more than just a transcription factor; in fact increased intracellular levels of the Myc/Max dimer seems to globally elevate the transcription rate of almost all genes normally expressed in that specific cellular system (Lin et al., 2012; Nie et al., 2012). Although this seems to be the case for actively transcribed genes, less clear is the role of the Myc/Max complexion repressed genes. However, these studies recognize that Myc can also determine repression of several hundred genes, the mechanism for that remains elusive or is simplistically relegated to a non-specified indirect function. Many studies regarding MYCN demonstrate that it is directly involved in such a phenomena and that Myc-mediated repression may involve direct interaction of Myc with many additional regulators of the transcription function. But why so many regulators? A possible explanation may be reached by looking at the dynamics through which the Myc/Max complex forms. There is no doubt that Myc and Max are “perfect, indissoluble partners” and that this is most likely what happens in normal conditions. The two proteins find each other and exert their function as a dimer complex. Nonetheless this situation is disturbed during oncogenesis, particularly when Myc starts to be up-regulated or even abnormally overexpressed as a consequence of chromosome translocations or gene amplification. In that case, the amount of Myc in the tumor cells strongly exceeds that of Max. Now in addition to increase the amount of the Myc/Max complex, the exceeding Myc may begin to establish uncontrolled liaisons with other nuclear components. The affinity for this components is probably lower as compared to that described for Max, but still strong enough to promote specific biochemical effects. There are few but compelling data that may fit with this model. For instance it has been found that Myc can repress transcription of TG2 by recruiting HDAC1 in the absence of Max. Secondly, there are cell lines such as the rat pheochromocytoma PC12 that express high levels of Myc but do not express Max. Recently it has been found that about 20% of human pheochromocytomas with high Myc carry mutations in the Max gene that cripple its ability to dimerize with Myc (Burnichon et al., 2012). Furthermore, genetic studies in *Drosophila* show that the fly larvae with a mutated, inactive Max can reach late stages of development whereas this is not possible when Myc is inactivated, suggesting that Myc may exert functions that go beyond its partnership with Max (Steiger et al., 2008). All together, these findings point to the repression function of MYC as an important determinant of oncogenesis. A better comprehension of these mechanisms and the identification of those nuclear proteins that engage MYCN during oncogenesis may highlight new druggable molecular targets that will be helpful to look for new anticancer drugs specifically focalized on defeating neuroblastoma.

## Conflict of Interest Statement

The authors declare that the research was conducted in the absence of any commercial or financial relationships that could be construed as a potential conflict of interest.
